# Supplementary material for: Epidemiology of Nosema spp. and the effect of indoor and outdoor wintering on honey bee colony population and survival in the Canadian Prairies
Source: PLoS One. 2021 Oct 25;16(10):e0258801. doi: 10.1371/journal.pone.0258801 (PMC8544878; doi:10.1371/journal.pone.0258801)
Supplement: S1 File — Supporting information for: Table A. Timeline for honey bee sampling and bee population estimates from May 2017 to April 2019 in Alberta colonies; Table B. Monthly climate normals data from weather stations near apiary sites; Table C. Monthly climate data from weather stations near apiary sites for 2017–2019. (DOCX) [file pone.0258801.s001.docx]

**Supporting Information for:**

**Epidemiology of *Nosema* spp. and the effect of indoor and outdoor wintering on honey bee colony population and survival in the Canadian Prairies.**

Rosanna N. Punko^1*^, Robert W. Currie^1^, Medhat E. Nasr^2^, Shelley E. Hoover^3^

^1^ Department of Entomology, University of Manitoba, Winnipeg, Manitoba, Canada

^2^ Alberta Agriculture and Forestry, Government of Alberta, Edmonton, Alberta, Canada (retired)

^3^ Department of Biological Sciences, University of Lethbridge, Lethbridge, Alberta, Canada

* Corresponding author

Email: punkor@myumanitoba.ca

**Table A.** **Timeline for honey bee sampling and bee population estimates from May 2017 to April 2019 in Alberta colonies.** Dates are presented as the average date for the sampling period.

| Date | Description |
| --- | --- |
| May 29-June 8, 2017 | Colony Equalization |
| June 14, 2017 | Sampling |
| July 15, 2017 | Sampling & Bee Population |
| July 28, 2017 | Sampling |
| August 12, 2017 | Sampling & Bee Population |
| August 26, 2017 | Sampling |
| September 10, 2017 | Sampling & Bee Population |
| September 27, 2017 | Sampling & Bee Population |
| April 25, 2018 | Sampling & Cluster Size |
| May 8, 2018 | Sampling & Bee Population |
| June 5, 2018 | Sampling & Bee Population |
| June 16, 2018 | Sampling |
| June 30, 2018 | Sampling & Bee Population |
| July 14, 2018 | Sampling |
| July 28, 2018 | Sampling & Bee Population |
| August 12, 2018 | Sampling |
| August 24, 2018 | Sampling & Bee Population |
| September 8, 2018 | Sampling |
| September 22, 2018 | Sampling & Cluster Size |
| April 5, 2019 | Sampling & Cluster Size |
| April 29, 2019 | Sampling (only South apiaries) |

**Table B.** **Monthly climate normals data from weather stations near apiary sites.**

Data provided by Environment and Climate Change Canada https://climate.weather.gc.ca (retrieved on June 2, 2020).

North Apiaries

**Station name:** Elk Island National Park

**Climate ID:** 3012275

**Location:** 53°40'58.000" N 112°52'05.000" W

**Elevation:** 716.2 m

South Apiaries

**Station name:** Vauxhall North

**Climate ID:** 3036690

**Location:** 50°10'57.080" N 112°07'19.040" W

**Elevation:** 760.0 m

**Table B.**

|  | Daily Average (℃) | | Daily Maximum (℃) | | Daily Minimum (℃) | | Days with Maximum Temperature above 10℃ | |
| --- | --- | --- | --- | --- | --- | --- | --- | --- |
| Apiary | North* | South** | North* | South** | North* | South** | North† | South‡ |
| January | -12.0 | -9.2 | -6.2 | -2.9 | -17.8 | -15.4 | 0.4 | 1.9 |
| February | -9.3 | -6.1 | -2.5 | 0.5 | -15.9 | -12.7 | 0.8 | 4.1 |
| March | -5.1 | -0.9 | 1.2 | 6.1 | -11.4 | -7.8 | 3.3 | 12.0 |
| April | 4.2 | 6.0 | 10.8 | 13.7 | -2.4 | -1.6 | 17.5 | 21.4 |
| May | 10.6 | 11.4 | 17.0 | 19.0 | 4.1 | 3.8 | 27.3 | 28.1 |
| June | 14.6 | 15.4 | 20.8 | 22.3 | 8.4 | 8.6 | 29.7 | 29.7 |
| July | 17.0 | 17.7 | 23.2 | 25.5 | 10.8 | 10.0 | 31.0 | 30.9 |
| August | 15.7 | 17.4 | 22.1 | 25.8 | 9.2 | 8.9 | 30.7 | 31.0 |
| September | 10.1 | 12.4 | 16.1 | 20.7 | 4.0 | 4.1 | 24.7 | 28.2 |
| October | 4.3 | 5.7 | 9.9 | 13.4 | -1.3 | -1.9 | 15.9 | 21.1 |
| November | -5.2 | -1.9 | -0.3 | 4.5 | -10 | -8.2 | 2.1 | 7.4 |
| December | -9.5 | -6.8 | -4.1 | -0.6 | -15.1 | -12.9 | 0.6 | 3.0 |
| Year | 3.0 | 5.1 | 9.0 | 12.3 | -3.1 | -2.1 | 183.8 | 218.8 |

*calculated using data from 1981-2007 (total 20 years, 8 years missing; ~98% of possible observations)

**calculated using data from 1987-2007 (total 21 years, 1 year missing; ~95% of possible observations)

†calculated using data from 1981-2007 (total 20 years, 8 years missing; 92.3% of possible observations)

‡calculated using data from 1988-2007 (total 20 years, 3 years missing; 87.4% of possible observations)

**Table C.** **Monthly climate data from weather stations near apiary sites for 2017-2019.**

Data provided by Alberta Agriculture and Forestry, Alberta Climate Information Service (ACIS) https://acis.alberta.ca (retrieved June 3, 2020).

North Apiaries

**Station name:** Elk Island National Park

**Climate ID:** 3012275

**Location:** 53°40'58.000" N 112°52'05.000" W

**Elevation:** 716.2 m

South Apiaries

**Station name:** Vauxhall CDA CS

**Climate ID:** 3036682

**Location:** 50°03'00.000" N 112°08'00.000" W

**Elevation:** 779.00 m

**Table C. continued next page.**

|  |  | Average Air Temperature (°C) | | Minimum Air Temperature (°C) | | Maximum Air Temperature (°C) | | Days with Maximum Temperature  above 10°C | |
| --- | --- | --- | --- | --- | --- | --- | --- | --- | --- |
| Year | **Apiary** | North | South | North | South | North | South | North | South |
| 2017 | May | 12.5 | 14.1 | -0.5 | 0.5 | 29.9 | 31.8 | 29 | 31 |
|  | June | 15.2 | 17.0 | 3.9 | 4.9 | 28.1 | 33.0 | 30 | 30 |
|  | July | 17.4 | 21.2 | 5.6 | 4.6 | 29.7 | 36.9 | 31 | 31 |
|  | August | 15.8 | 18.7 | 4.7 | 4.9 | 28.4 | 35.1 | 31 | 31 |
|  | September | 10.9 | 13.3 | -0.6 | -3.8 | 30.1 | 35.4 | 25 | 28 |
|  | October | 4.1 | 6.2 | -5.9 | -7.8 | 20.2 | 24.1 | 15 | 20 |
|  | November | -8.1 | -3.9 | -21.6 | -24.9 | 6.3 | 16.5 | 0 | 4 |
|  | December | -8.6 | -7.5 | -34.6 | -35.2 | 9.2 | 14.9 | 0 | 5 |
| 2018 | January | -10.8 | -7.9 | -35.4 | -39.8 | 9.0 | 8.4 | 0 | 0 |
|  | February | -13.4 | -14.6 | -32.0 | -38.5 | 7.2 | 4.8 | 0 | 0 |
|  | March | -6.3 | -7.0 | -26.0 | -29.2 | 10.7 | 6.4 | 1 | 0 |
|  | April | 0.3 | 1.9 | -26.6 | -20.0 | 27.5 | 28.4 | 12 | 16 |
|  | May | 14.3 | 15.3 | -1.0 | 0.0 | 29.3 | 31.1 | 30 | 31 |
|  | June | 15.9 | 16.9 | 3.9 | 4.4 | 29.8 | 31.5 | 30 | 30 |

**Table C. continued…**

|  |  | Average Air Temperature (°C) | | Minimum Air Temperature (°C) | | Maximum Air Temperature (°C) | | Days with Maximum Temperature above 10°C | |
| --- | --- | --- | --- | --- | --- | --- | --- | --- | --- |
| Year | **Apiary** | North | South | North | South | North | South | North | South |
| 2018 | July | 17.2 | 19.1 | 5.4 | 6.1 | 31.4 | 35.3 | 31 | 31 |
|  | August | 15.4 | 17.7 | 1.8 | 2.4 | 34.6 | 39.9 | 31 | 31 |
|  | September | 5.7 | 9.3 | -3.1 | -3.6 | 25.7 | 31.7 | 15 | 23 |
|  | October | 2.9 | 4.9 | -6.4 | -7.1 | 24.3 | 23.5 | 14 | 22 |
|  | November | -3.9 | -0.7 | -19.3 | -19.9 | 11.4 | 14.2 | 1 | 10 |
|  | December | -7.9 | -3.2 | -28.4 | -21.7 | 7.2 | 11.5 | 0 | 3 |
| 2019 | January | -9.2 | -4.5 | -31.1 | -25.8 | 8.8 | 13.7 | 0 | 2 |
|  | February | -22.3 | -20.3 | -43.9 | -35.0 | -1.5 | 1.3 | 0 | 0 |
|  | March | -5.3 | -4.2 | -39.8 | -40.2 | 18.2 | 18.2 | 2 | 11 |
|  | April | 4.0 | 6.9 | -6.6 | -6.3 | 20.6 | 22.6 | 15 | 26 |
|  | May | 10.7 | 10.7 | -4.3 | -5.0 | 29.1 | 30.1 | 25 | 28 |
